# Temporal Dynamics of Diffusion Metrics in Early Multiple Sclerosis and Clinically Isolated Syndrome: A 2-Year Follow-Up Tract-Based Spatial Statistics Study

**DOI:** 10.3389/fneur.2019.01165

**Published:** 2019-11-05

**Authors:** Ruth Schneider, Erhan Genç, Christian Ahlborn, Ralf Gold, Carsten Lukas, Barbara Bellenberg

**Affiliations:** ^1^Department of Neurology, St. Josef Hospital, Ruhr University Bochum, Bochum, Germany; ^2^Department of Biopsychology, Institute of Cognitive Neuroscience, Ruhr University Bochum, Bochum, Germany; ^3^Institute of Neuroradiology, St. Josef Hospital, Ruhr University Bochum, Bochum, Germany

**Keywords:** multiple sclerosis (MS), clinically isolated syndrome (CIS), diffusion tensor imaging (DTI), tract-based spatial statistics (TBSS), fractional anisotropy (FA), radial diffusivity (RD)

## Abstract

**Background:** Tract-based spatial statistics (TBSS) is suitable for the assessment of voxel-wise changes in fiber integrity in WM tracts in the entire brain. Longitudinal TBSS analyses of early multiple sclerosis (MS) using 3 Tesla magnetic resonance imaging (MRI) are not common.

**Objective:** To characterize microstructural WM alterations at initial diagnosis in clinically isolated syndrome (CIS) and early MS at baseline and longitudinally over 2 years.

**Methods:** DTI (Diffusion tensor imaging) at 3 Tesla was used to evaluate 106 therapy-naive patients with CIS or definite MS at baseline and at 1-year (*N* = 83) and 2-year (*N* = 43) follow-up compared to healthy controls (HC, *N* = 49). TBSS was used for voxel-wise analyses of the DTI indices of fractional anisotropy (FA) and radial, mean, and axial diffusivity (RD, MD, AD) for cross-sectional and longitudinal comparisons. Mean values of FA, RD, and cluster voxel numbers were extracted from significant clusters using an atlas-based approach. Correlations with disability (EDSS) were calculated for FA and RD changes related to affected brain regions.

**Results:** Reductions in FA compared to HC were found at baseline in patients with CIS and RRMS and involved most supra- and infratentorial WM tracts. In the cerebellum and cerebral peduncles, these changes negatively correlated with EDSS after 2 years. FA changes in patients with CIS and RRMS evolved in the second year, particularly in the descending projection pathways and the cerebellum, and were significantly associated with EDSS. RD alterations compared to HC were undetectable in patients at baseline but were observed after 1 year and were exacerbated during the second year in all major supratentorial WM tracts, the corpus callosum, and the cerebellum. FA did not change between baseline and year 1 follow-up, but longitudinal investigation between the first and second year revealed combined dynamic FA and RD changes in the corpus callosum and corona radiata.

**Conclusion:** TBSS of diffusion metrics at initial diagnosis and at 2-year follow-up showed microstructural WM pathology and associations between FA reduction and future disability, respectively. Combined longitudinal changes in FA and RD occurred in specific structures, where RD increases likely reflected progressing axonal degeneration. The distinct temporal dynamics of FA and RD, implying constancy during the first year, supports early therapeutic intervention for CIS and RRMS.

## Introduction

Multiple sclerosis (MS) is a chronic disease of the central nervous system (CNS) characterized by demyelination and axonal injury. Conventional magnetic resonance imaging (MRI) is a very sensitive technique for detecting focal MS-related macroscopic lesions but suffers from a lack of histopathological specificity. Several MRI studies using non-conventional MRI techniques such as DTI have confirmed that MS-related damage also affects white matter (WM) at the microscopic level (1). As such, evaluation of microstructural changes in the brain beyond the locations of MS lesions using MRI techniques has become critical for the evaluation of early MS (2–4). DTI has shown decreased fractional anisotropy (FA) and increased mean diffusivity (MD) in areas of macroscopically normal brain tissues in patients with MS, indicating subtle, diffuse injury, with increased mobility of water molecules and disruption of tissue architecture (5). Radial diffusivity (RD) and axial diffusivity (AD), which are additional DTI metrics, have been suggested as markers of myelin and axonal damage, respectively (6). Recent studies have demonstrated the power of radial diffusivity for clinical-morphological correlations in MS (1, 7, 8). DTI has been used in various MS studies to identify and explore anatomical connectivity, resulting in improved understanding of the mechanisms of MS progression (9, 10).

Several methods for DTI quantification have been introduced, ranging from regional assessment, such as region of interest (ROI)-based analysis, to more detailed voxel-based statistical analysis exploring total brain tissue, such as tract-based spatial statistics (TBSS). TBSS is a non-hypothesis-driven technique allowing for voxel-wise assessment of changes in diffusion metrics without identifying a specific anatomical target (11). This technique has been used cross-sectionally to assess white matter changes in a range of MS disease subgroups (12, 13) and disabilities (1, 14). However, though TBBS has been used to evaluate longitudinal changes in MS with longer disease duration (15–18), there is still a lack of longitudinal DTI studies in early MS or CIS patients. Thus, the aim of the current study was to investigate diffusion metrics longitudinally using TBSS based on 3 Tesla MRI to evaluate a large group of patients at disease onset to study the temporal dynamics of early microstructural WM changes in MS during a 2-year follow-up study.

## Methods

### Patient and Healthy Controls

**Figure 1:**
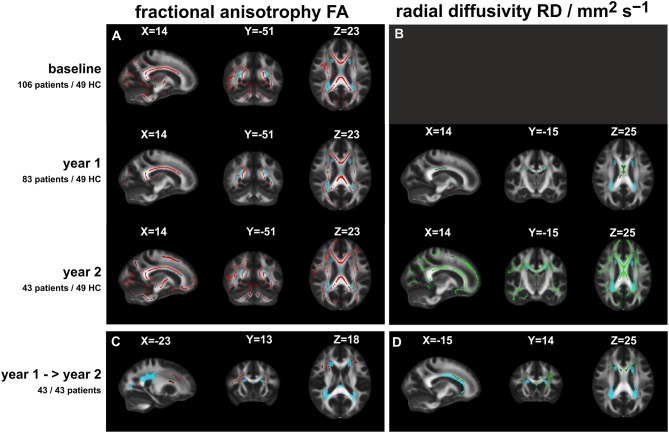
Significant FA or RD differences between patients and healthy controls (TBSS analyses: *t*-tests including age and sex as nuisance variables; with correction for multiple comparisons by TFCE cluster enhancement at a significance threshold of *p* < 0.01). **(A)** (Left column): fractional anisotropy FA (red overlay) at three time points; **(B)** (right column): radial diffusivity RD (green overlay) at FU1 and FU2 (no significant differences at baseline). Lowest row: comparison of FA **(C)** and RD **(D)** between patients at year 1 and at year 2. The light blue overlays depict the lesion distribution (threshold: 0.1) at the specific time points; X, Y, Z: MNI coordinates of slice positions.

A total of 106 patients with either clinically isolated syndrome (CIS, *N* = 51) or early RRMS (*N* = 55) were enrolled from an ongoing longitudinal study of the German Competence Network Multiple Sclerosis (KKNMS). These patients were included and examined at a single center between 2011 and 2015. According to the study design, the enrolled patients at study entry were therapy-naive to disease-modifying drugs and had either a diagnosis of CIS with a high risk of conversion to MS within 6 months or early definite MS where it was <24 months after the onset of symptoms (19). The patients were compared to an age- and sex-matched healthy control group (HC, *N* = 49). Longitudinal information was collected in a subset of patients after 1 year (FU1: follow-up 1) and 2 years (FU2: follow-up 2). Eighty-three patients underwent FU1 (78%), and 43 underwent baseline, FU1, and FU2 follow-up (41%) investigation. The follow-up setting is illustrated in Figure 1. All patients underwent neurological examinations at baseline and follow-up, including the Expanded Disability Status Scale (EDSS) (20). The study was approved by the local ethics committee of Ruhr-University Bochum (Approval No. 3714-10), and all patients provided written informed consent. Details of the study population are presented in Table 1.

**Table 1:** Demography of the participants at baseline and follow-up; mean ± standard deviations for age and disease duration, median (interquartile range) for lesion volume, median [range] for EDSS; classification into CIS or RRMS at follow-up according to the disease type at baseline.

	**All patients**	**CIS**	**RRMS**	**HC**	**Significance**
N (baseline)	106	51	55	49	
Female/male	68/38	32/19	36/19	32/17	n.s.^a^
N (year 1)	83	36	47	n.a.	
Female/male	51/32	21/15	30/17		n.s.^a^
N (year 2)	43	19	24	n.a.	
Female/male	24/19	10/9	14/10		n.s.^a^
Age/years at baseline	37 ± 11	36 ± 11	37 ± 11	42 ± 14	All patients/HC: *p* = 0.027^b^; CIS/RRMS/HC: *p* = 0.047^b^
Months since symptom onset (baseline)	12 ± 10	10 ± 10	14 ± 11	n.a.	CIS/RRMS: *p* = 0.018^b^
Lesion volume (ml) at BL; median (IQR) (*N* = 106)	2.1 (0.9–5.3)	2.1 (0.9–5.9)	2.2 (1.2–5.2)	n.a.	n.s.^c^
Lesion volume (ml) at BL^*^; median (IQR) (**N=43**)	*3.0 (1.8*–*9.0)*	*3.7 (2.0*–*15.3)*	*2.2 (1.6*–*5.1)*	*n.a*.	*n.s.^c^*
Lesion volume (ml) year 1^*^; median (IQR) (**N=43**)	*3.3 (1.8–7.9)*	*4.3 (2.0–8.7)*	*2.3 (1.7–5.3)*	*n.a*.	*n.s.^c^;* *n.s.^d^ (FU1-BL)*
*Lesion volume (ml) year 2; median (IQR) (**N=43**)*	*3.2 (1.8–7.8)*	*5.2 (2.2–8.7)*	*2.1 (1.6–5.1)*	*n.a*.	*n.s.^c^;* *n.s.^d^ (FU2-BL, FU2-FU1)*
EDSS baseline; median (range)	1.5 [0.0. 6.5]	1.5 [0.0. 4.0]	1.5 [0.0. 6.5]	n.a.	EDSS change (FU2-BL)^d^: all patients: *p* = 0.059, CIS: *p* = 0.044, RRMS: *p* = 0.410
EDSS year 1; median (range)	1.5 [0.0. 6.5]	1.5 [0.0. 5.0]	1.5 [0.0. 6.5]	n.a.	
EDSS year 2; median (range)	2.0 [1.0. 5.0]	2.0 [1.0. 5.0]	2.0 [1.0. 5.0]	n.a.	

n.s., not significant; IQR, interquartile range;

* (italic): Lesion volume for subgroups scanned at BL, FU1 and FU2, bold printed N, number of patients in follow-up lesion analysis; significance of group differences:

a *Chi-square test*,

b *univariate ANOVA with post-hoc pairwise comparisons*,

c *Mann–Whitney U-test between CIS and RRMS*,

d *paired Friedmann's rank test*.

### MR Imaging

Imaging was performed using a single 3T scanner (Achieva; Philips, The Netherlands) without changes to the hardware or software during follow-up investigation. A standardized imaging protocol was used, including a single-shot 2D echo planar imaging (EPI) sequence for diffusion tensor imaging (DTI) [EPI single shot, 50 axial slices, 2.5 mm slice thickness, field of view 320 × 240, in-plane resolution: (2.5 × 2.5) mm^2^, repetition time (TR), echo time (TE)/ms 7000/90, flip angle:90°, 32 gradient directions, maximum B-factor: 900], an isotropic 3D fluid-attenuated inversion recovery (FLAIR) sequence for lesion quantification (3D FLAIR, 170 sagittal slices, field of view: 240 mm, resolution: (1 × 1 × 1) mm^3^, TR, TE, inversion time (TI)/ms: 4,800/286/1,650, turbo factor: 182, acquisition time: 6′30″ min), and a structural isotropic T1-weighted 3D sequence (T1 fast field echo; 180 sagittal slices; FOV: 240 mm × 240 mm; voxel size: 1 mm × 1 mm × 1 mm; TR, TE, TI/ms: 10/4.6/1000; flip angle: 8°, turbo factor: 164; acquisition time: 6′00″min).

### TBSS

Voxel-wise statistical analysis of DTI data was performed using Tract-Based Spatial Statistics (TBSS) version 1.2 (11), part of the FSL software package (21) (https://fsl.fmrib.ox.ac.uk). FSL software tools were used for eddy-current corrections (registration-based “eddy_correct” tool), brain extraction, and subsequent creation of FA images by fitting a tensor model to the raw diffusion data using the FSL-FDT tools (FMRIB's Diffusion Toolbox). FA data were then warped to a 1 × 1 × 1 mm^3^ standard space target image (FMRIB58_FA) using non-linear registration tools followed by affine registration to the MNI152 space. Next, the mean FA image was created and thinned to create a mean FA skeleton that represented the centers of all tracts common to the group (threshold = 0.2). The aligned FA data were then projected onto this skeleton, and the resulting skeletonized data were used to perform voxel-wise cross-subject statistics. Furthermore, skeletonized images of the mean diffusivity (MD), radial diffusivity (RD), and axial diffusivity (AD) were generated using TBSS tools with the same registration as was calculated for FA. Global DTI metrics for each participant were extracted by averaging FA, MD, RD, and AD across the entire white matter (WM) skeleton using fslstats. The TBSS results were visualized using the FSLeyes viewer.

#### Between-Group Comparisons

Voxel-wise statistics for the skeleton voxels were calculated for cross-sectional comparison of healthy controls to all patients at baseline and at years 1 and 2 follow-up. At baseline, additional subgroup comparisons of patients with CIS and RRMS with controls were assessed. Subgroup comparisons were not performed at the 1- and 2-year follow-ups because of smaller group sizes. We used the “randomize” approach in FSL for non-parametric voxel-wise statistical analysis (5,000 permutations) with two-sample unpaired *t*-tests (age and sex as nuisance variables) for between-group analyses of each DTI metric (FA, MD, RD, and AD).

#### Longitudinal Analyses

TBSS was also used to analyze the comparisons between patients at baseline and at years 1 and 2 follow-up relative to HC. Comparisons between BL and FU1 and between FU1 and FU2 were performed using FSL randomize for repeated measures (ANOVA with age and sex as nuisance variables) for each DTI metric (FA, MD, RD, and AD).

In the cross-sectional and longitudinal analyses, clusters with significantly altered DTI parameters were identified using threshold-free cluster enhancement (the TFCE option for randomize). Because the option for repeated measures in randomize only allows for pairwise comparisons, we defined a more conservative significance threshold of *p* < 0.01 (instead of the commonly used level of *p* < 0.05) to minimize the probability of type-1 errors in the longitudinal analyses between baseline, years 1, and 2. To ensure comparability, we also chose the same significance level of *p* < 0.01 for the cross-sectional analyses.

#### Extraction of Quantitative DTI Parameters

Sets of regions of interest (ROI) were defined according to the white matter regions showing significant group differences in the cross-sectional or longitudinal analyses (clusters). Means and standard deviations of FA, RD, MD, AD, and cluster voxel numbers were extracted from the relevant brain regions using the image statistics utilities in FSL (FSLUTILS: fslmaths, fslmeants, fslstats) of different DTI-based brain white matter label atlases included in FSL (JHU-ICBM_labels _1 mm (22) and the probabilistic cerebellar atlas (Cerebellum-MNI_fnirt_1 mm) (23). In each analysis, binary mask images of the significant areas on the skeleton were generated using the FSLeyes viewer and labeled by multiplication with the atlas file using fslmaths. The number of significant voxels on the skeleton in each labeled brain region was calculated using fslstats. The aligned FA, MD, RD, and AD maps were then multiplied with the labeled mask images, and the fslmeants utility was used to calculate the mean and standard deviation of diffusion metrics in the significant labeled brain regions.

### Lesion Quantification

Total FLAIR lesion volume was calculated for each patient using the lesion prediction algorithm in LST toolbox version 2.0.15 (https://www.statistical-modelling.de/lst.html) for LST, a toolbox extension of SPM12 (24). During the lesion segmentation procedure, the FLAIR images and resulting lesion maps were coregistered to the 3D-T1-weighted series. The T1-weighted series were registered and normalized to the DARTEL template in MNI space using the preprocessing procedures of the CAT12 segmentation tools (CAT12, version R1165, http://www.neuro.uni-jena.de/hbm2016/GaserHBM2016.pdf).

The deformation fields of the T1 transformations were applied to the individual lesion maps to transform them to the common MNI-space using the SPM12 Normalize (write) function. Mean lesion maps of patient subgroups were calculated using the SPM12 image calculator tool. The visual representation of the mean lesion maps in the FSLeyes viewer was thresholded at a level of 0.1, showing regions were at least 10% of the patients had FLAIR lesions.

### Statistics

SPSS software (IBM Corp. Released 2016. IBM SPSS Statistics for Windows, Version 24.0. Armonk, NY: IBM Corp.) was used for further statistical analyses of quantitative results. Comparisons of demographic data and quantitative DTI results between groups were assessed by univariate ANOVA (three groups or more), Mann–Whitney-*U*-tests (two groups), or Chi-squared tests (for number of participants). Longitudinal EDSS changes were analyzed using paired Friedmann's rank tests. Spearman rank correlation analyses were used to determine the association between DTI metrics in significant clusters on the WM skeleton and EDSS. To account for the risk of false-positive findings due to multiple testing of correlations with EDSS in 30 different atlas-based regions, we applied a Bonferroni correction threshold for significance of *p* < 0.002 (calculated as 0.05/30). These correlation analyses were assessed at baseline separately for the RRMS and CIS subgroups and at follow- up for those patients who experienced worsened or improved EDSS, which was defined as an EDSS increase or decrease by 0.5, during the 2-year follow-up period.

## Results

### Demographic, Clinical, and Volumetric Data (Table 1)

At baseline, there were no significant differences in the age or proportion of each sex between the CIS and RRMS groups. Based on the study design, the disease duration since symptom onset was longer in the RRMS group by definition. The median EDSS was similarly low in both subgroups at baseline (median 1.5). After study entry, 83 patients (80%) started a medication. The most common medications were Glatirameracetat (Copaxone) (32%), Interferon beta-1a i.m. (Avonex) (20%), Interferon beta-1a s.c. (Rebif) (17%), and Interferon beta-1b (Betaferon) (9.5%).

Total brain lesion load at baseline was low, with no significant differences between the CIS and RRMS groups (*p* = 0.985). During follow-up, there was no significant increase in brain lesion load in the entire patient group and in CIS and RRMS regarded separately, neither among all patients who were scanned at baseline and year 1 (*N* = 83) nor in the subgroups of patients who later reached follow-up 2 (year 2, *N* = 43). Additionally, changes between years 1 and 2 were not significant in any subgroup (Table 1).

The local distribution of the T2-FLAIR lesions was mainly restricted to periventricular and callosal regions. Matching of the lesion distribution with the skeleton of the white matter tracts showed small overlapping areas including parts of the corpus callosum, the bilateral posterior thalamic radiation (incl. optic radiation), the anterior and superior corona radiata, and the sagittal stratum. Figure S1 shows an overlay of the mean lesion distribution maps (blue: at baseline; red: FU2) of the patient subgroup who received year 2 follow-up overlain on a T1-template and the mean FA skeleton. Ony subtle lesion volume increases mainly located in the posterior corona radiata can be seen.

During the follow-up interval, there was a significant increase in EDSS in the group of patients classified as CIS at baseline, but the increase was not significant in the RRMS group. Comparison of baseline and year 2 EDSS showed that 32 of 43 MS patients (74.4%) experienced EDSS-changes (12/43 showed decreased EDSS, and 20/43 showed increased EDSS). Eleven patients had stable EDSS results at FU2.

### TBSS Results

#### Global Changes in DTI Parameters

We calculated global DTI measures by averaging FA, MD, RD, and AD across the entire WM skeleton. At baseline, significantly lower FA and higher MD and RD were observed in patients compared to controls, while CIS- RRMS differences were not significant (Table 2).

**Table 2:** Global DTI measures at baseline (FA, MD, RD, AD) throughout the white matter skeleton (mean ± standard deviation).

**Global skeleton DTI metrics Mean ± SD**	**All patients**	**CIS**	**RRMS**	**HC**	**P_**ANOVA**_**	**P_**patients-HC**_**
	***N* = 106**	***N* = 51**	***N* = 55**	***N* = 49**		
FA	0.608 ± 0.023	0.609 ± 0.024	0.606 ± 0.023	0.616 ± 0.017	0.072	0.014
MD (10^−3^ mm^2^/s)	0.394 ± 0.011	0.399 ± 0.017	0.400 ± 0.016	0.394± 0.011	0.083	0.013
RD (10^−3^ mm^2^/s)	0.241 ± 0.021	0.240 ± 0.021	0.242 ± 0.020	0.234 ± 0.014	0.085	0.016
AD (10^−3^ mm^2^/s)	0.717 ± 0.016	0.717 ± 0.016	0.717 ± 0.016	0.714 ± 0.013	0.505	0.242

*Significance of group differences between all patients and HC assessed using Student's t-tests and between CIS, RRMS, and HC using univariate ANOVA (Sidak correction for multiple comparisons)*.

*SD, standard deviation; N, number of participants; FA, fractional anisotropy; MD, mean diffusivity; RD, radial diffusivity; AD, axial diffusivity*.

#### Voxel-Wise DTI Analysis

Voxel-wise TBSS analysis showed local clusters of significantly reduced FA in patients compared to controls at baseline and at FU1, with FA reduced further at FU2 throughout most of the major brain white matter tracts, including the cerebellum (Figure 1, left row, red overlay). Similar patterns of increased MD were observed at baseline, FU1, and FU2, with the exception of a lack of infratentorial involvement in MD (not shown). In contrast, voxel-wise RD was not significantly different at baseline between patients and controls, but at FU1, and more so at FU2, RD was increased mostly in the corpus callosum at years 1 and at 2 and was additionally increased significantly at FU2 in the frontal, temporal, and occipital tracts and parts of the cerebellum (Figure 1, right row, green overlay). AD was significantly increased in the corpus callosum and the pyramidal tracts at baseline but not at FU1 or FU2, possibly due to the smaller patient groups at these time points (not shown).

No significant changes were observed in the longitudinal analysis between patients at baseline and FU1, neither in the entire group of patients who received the follow-up at year 1 (*N* = 83) nor in the subgroup of patients who were scanned at FU1 and FU2 (*N* = 43). The longitudinal comparison between patients at years 1 and 2 showed significant decreases in FA and significant increases in RD, primarily in the frontal and callosal WM tracts, and to a lesser extent in the internal capsule (see Figure 1, lowest row). Similar, but smaller, callosal clusters of increased MD were observed.

The light blue overlays in Figure 1 show the mean lesion distribution maps at the specific time points. The overlap with the DTI alterations was small and was restricted to parts of the bilateral posterior thalamic radiation (incl. optic radiation), the anterior and superior corona radiata, and the sagittal stratum.

Table 3 summarizes global changes in DTI metrics within brain white matter tracts. In subsequent analyses, we evaluated FA and RD since longitudinal inter-patient changes were mainly restricted to these parameters.

**Table 3:** Overall DTI changes (FA, MD, RD, AD) in the WM tracts at different timepoints, comparing patient groups with HC and comparing patients at follow-up 1 and at follow-up 2 (↑, increased value; ↓, decreased value; -, no significant changes).

**Time of MRI**	**Numbers of participants**	**Comparison**	**FA**	**MD**	**RD**	**AD**
Baseline	49/106	HC - all patients	↓	↑	–	↑
FU1	49/83	HC - all patients	↓	↑	↑	–
FU2	49/43	HC - all patients	↓	↑	↑	–
FU1 - FU2	43/43	all patients - all patients	↓	↑	↑	–
						

#### Atlas-Based Extraction of Involved Regions

##### Baseline: CIS and RRMS compared to healthy controls

Areas with significant reductions in FA compared to HC were observed in the CIS and RRMS groups. Specifically, all white matter tracts had decreased FA compared to HC in both subgroups, and FA was decreased compared to the HC group in the brainstem and cerebellum, primarily in the RRMS group. No significant differences were observed in the DTI metrics of any brain areas between the CIS and RRMS groups.

However, quantitative atlas-based extraction of affected regions showed specific differences between the affected regions in the comparison between the CIS and HC groups and in that of the RRMS and HC groups. Reduced FA compared to HC was observed in both MS subtypes, but the decrease was more pronounced in the RRMS group, as demonstrated by higher numbers of involved voxels in significantly affected WM-tracts and by lower FA values in the RRMS group compared to the CIS group (see Table 4). Table 4 lists only those brain structures in which the number of affected voxels in the RRMS group was more than 50 voxels higher than that in the CIS group [external capsule (left), superior corona radiata (right), and superior longitudinal fasciculus (right) (>500 voxel-differences)]. The posterior thalamic radiation (right+left) and body of the corpus callosum were similarly affected in the CIS and RRMS groups but to a slightly greater extent in the RRMS group. Only a few regions were exclusively affected in the RRMS group: the fornix (column and body, listed in Table 4) and the left posterior limb of the IC (37 voxels), middle cerebellar peduncle (29 voxels), left inferior cerebellar peduncle (24 voxels), and left cerebral peduncle (19 voxels). These regions are not summarized in Table 4 due to their smaller affected voxel numbers. Cerebellar affection compared to HC at baseline was observed merely in the entire patient group, as evidenced by significantly smaller FA values in anterior lobules I–IV and V (Table 5).

**Table 4:** FA changes in different anatomical regions in CIS or RRMS patients at baseline in comparison to healthy controls.

**Region**		**CIS (*****N*** **=** **55)**		**RRMS (*****N*** **=** **55)**		
	**MNI coordinates (X, Y, Z)**	**Voxels in cluster**	**FA (mean ± SD)**	**Voxels in cluster**	**FA (mean ± SD)**	**Voxel difference**
External capsule L	(−31, 5, −8)	25	0.81 ± 0.08	948	0.66 ± 0.07	923
Superior corona radiata R	(27, −17, 24)	326	0.83 ± 0.04	836	0.78 ± 0.04	510
Superior longitudinal fasciculus R	(37, −23, 30)	193	0.75 ± 0.04	703	0.75 ± 0.05	510
Posterior corona radiata R	(26, −27, 27)	439	0.76 ± 0.04	721	0.75 ± 0.05	282
Fornix (column and body)	(1,−10, 16)	–	–	246	0.61 ± 0.08	246
Retrolenticular part of IC R	(29, −25, 6)	417	0.84 ± 0.04	657	0.84 ± 0.04	240
Anterior corona radiata R	(21, 37, 1)	423	0.76 ± 0.07	649	0.77 ± 0.06	226
Cingulum (hippocampus) L	(−22, −27, 82)	3	0.63 ± 0.08	164	0.75 ± 0.07	161
Cingulum (cingulate gyrus) R	(7, 6, 33)	21	0.82 ± 0.05	164	0.79 ± 0.05	143
Posterior thalamic radiation incl. optic radiation L	(−32, −62, 1)	884	0.83 ± 0.04	1,008	0.83 ± 0.05	124
Superior corona radiata L	(−26, −17, 24)	384	0.77 ± 0.06	497	0.75 ± 0.05	113
Retrolenticular part of IC L	(−27, −25, 6)	134	0.80 ± 0.05	237	0.84 ± 0.04	103
Posterior thalamic radiation incl. optic radiation R	(33, −62, 1)	873	0.84 ± 0.04	963	0.84 ± 0.04	90
Fornix (cres)/L	(−27, −26, −6)	117	0.82 ± 0.06	204	0.80 ± 0.05	87
Body of corpus callosum	(−5, −26, 25)	2707	0.90 ± 0.04	2793	0.90 ± 0.04	86
Sagittal stratum (include inf. long.fasc. and inf. front.-occip.fasc.) L	(−41, −29, −12)	277	0.80 ± 0.04	352	0.82 ± 0.04	75
Anterior corona radiata L	(−20, 37, 1)	90	0.75 ± 0.08	159	0.75 ± 0.08	69
Cerebral peduncle R	(15, −19, −12)	202	0.92 ± 0.03	266	0.95 ± 0.03	64
Fornix (cres)/R	(28, −26, −6)	144	0.82 ± 0.06	208	0.80 ± 0.06	64
Posterior corona radiata L	(−25, −27, 27)	529	0.74 ± 0.04	582	0.72 ± 0.05	53
Cingulum (hippocampus) R	(24, −25, −19)	59	0.72 ± 0.09	112	0.74 ± 0.07	53
External capsule R	(32, 5, −8)	378	0.73 ± 0.07	301	0.71 ± 0.07	−77
Splenium of corpus callosum	(5, −37, 16)	1,589	0.95 ± 0.03	1,413	0.94 ± 0.03	−176

*Mean and standard deviations of FA in clusters in which significant differences between patients and HC were found are shown for each group. The sizes of significant clusters for each region are reported as voxel numbers in cluster. An increase of voxel number represents larger cluster sizes in RRMS compared to CIS*.

*FA, fractional anisotropy; SD standard deviation; HC, healthy controls; N, number of participants; L, left hemisphere; R, right hemisphere; MNI, coordinates: centers of involved structures*.

**Table 5:** Significant FA changes in the cerebellar regions of patients at baseline in comparison to healthy controls.

**Region**			**FA (mean** **±** **SD)**	
**Cerebellum lobules (coordinates: x, y, z)**	**MNI coordinates (X, Y, Z)**	**Voxel no. in cluster**	**All patients *N* = 106**	**HC *N* = 49**
**Patients compared to HC**				
Left I–IV	(−6, −46, −18)	430	0.35 ± 0.03	0.37 ± 0.03
Right I–IV	(7, −46, −17)	350	0.37 ± 0.03	0.39 ± 0.03
Left V	(−16, −51, −20)	306	0.39 ± 0.03	0.40 ± 0.02
Right V	(13, −55, −17)	280	0.37 ± 0.02	0.39 ± 0.02
Left VI	(−14, −69, −22)	399	0.35 ± 0.03	0.36 ± 0.02

*Mean and standard deviations of FA in significant clusters are shown for each group. The sizes of significant clusters for each atlas region are reported as voxel numbers in cluster*.

*SD, standard deviation; HC, healthy controls; FA, fractional anisotropy; N, number of participants*.

**Figure 2:**
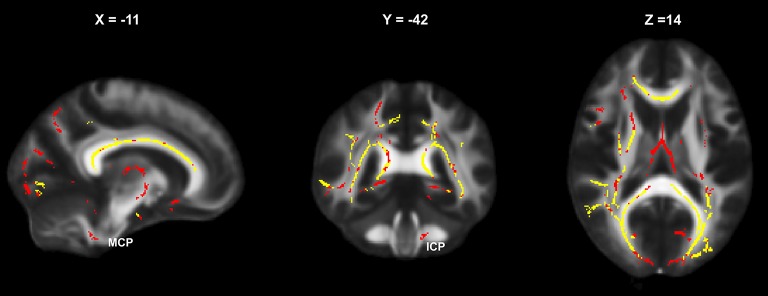
Baseline areas of significantly altered FA (relative to HC) in the CIS and RRMS subgroups: yellow, simultaneous affection in CIS and RRMS; red, altered only in RRMS (MCP, middle cerebellar peduncle; ICP, inferior cerebellar peduncle).

An overview of the FA alterations at baseline in CIS and RRMS is presented in Figure 2.

##### Longitudinal follow-up: patients at FU1 and FU2 compared to healthy controls at baseline

The significant local FA reductions compared to HC in patients at baseline were also observed at FU1 and FU2. In addition, RD was significantly increased compared to HC in the patient groups at FU1 and FU2 but not at baseline.

**Figure 3:**
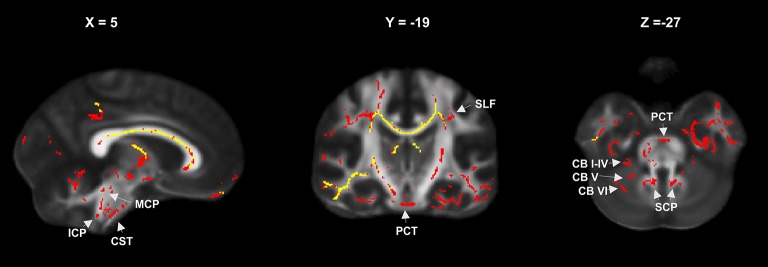
FA reduction relative to controls in the entire patient group at year 1 (yellow) and year 2 (red) showing progressive involvement of infratentorial and cerebellar fiber tracts (MCP, mean cerebellar peduncle; SCP, superior cerebellar peduncle; ICP, inferior cerebellar peduncle; CB I–IV, CB V, CB VI, cerebellar lobules; PCT, pontine crossing tracts; CST, corticospinal tracts; SFC, superior longitudinal fasciculus).

Differences in FA and RD changes were further quantified by atlas-based extraction of DTI metrics in the affected regions. Comparison of WM tracts with significantly decreased FA compared to HC in patients at FU1 and FU2 showed conclusive progressive involvement of descending infratentorial and cerebellar fiber tracts. Structures that did not exhibit changes in FA compared to HC in patients at FU1 but were significantly different at FU2 are summarized in Figure 3. Details are provided in Table S1. This effect was most pronounced in the middle cerebellar peduncle, corticospinal tract, and pontine tracts (pontine crossing tracts and left and right medial lemnisculus) and also occurred in the bilateral inferior and superior cerebellar peduncles and the cerebellum (anterior lobules I–IV, V, and the left and vermal lobule VI) (Table S1 and Figure 3).

**Figure 4:**
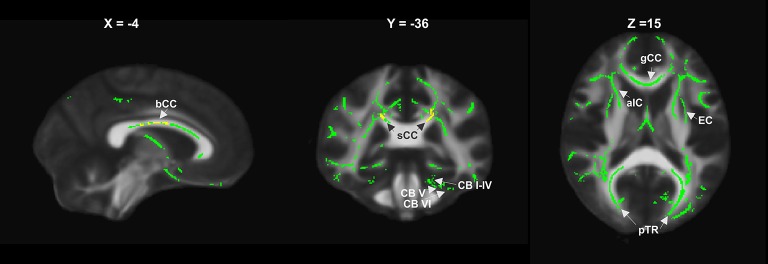
RD increase relative to controls in the entire patient group at year 1 (yellow) and year 2 (green), showing widespread supratentorial and partly cerebellar involvement (bCC, sCC, gCC body, splenium and genu of corpus callosum; EC, external capsule; aIC, anterior internal capsule; pTR, posterior thalamic radiation; CB I–IV, CB V, CB VI, cerebellar lobules).

Changes in RD compared to HC at FU1 were restricted to a few regions (the body and splenium of the corpus callosum and the bilateral superior and posterior corona radiata). At FU2, there was a considerable increase in the number of affected regions, with significant RD alterations in patients compared to HC (Figure 4). Regions with the largest voxel differences (>500 voxels more at FU2 than at FU1) were the genu, body, and splenium of the corpus callosum, the anterior, superior, and posterior corona radiata (right and left), the posterior thalamic radiation (right and left), the external capsule (right and left), and the superior longitudinal fasciculus (right). Furthermore, cerebellar WM tracts were also affected at FU2 but not at FU1. Details are shown in Table S2 and Figure 4 summarizes the differences between RD involvement at years 1 and 2.

The number of patients who received the FU2 MRI (*N* = 43) was considerably smaller than that at FU1 (*N* = 83), but the effects on FA and RD were much stronger at FU2 than at FU1.

##### Longitudinal DTI analysis and TBSS results comparing patients at FU1 and FU2

The TBSS between-group analysis of patients at FU1 compared to patients at baseline showed no significant changes in any diffusion metric, neither in the entire group of patients who received FU1 (*n* = 83) nor in the subgroup who also received FU2 (*n* = 43). In contrast, the comparison of patients at FU2 (*N* = 43) with patients at FU1 showed areas with significantly decreased FA and increased RD values (Figures 1C,D).

Quantitative atlas-based analysis showed significant FA differences between FU1 and FU2, mainly in the body of the corpus callosum (237 voxels) and in the anterior corona radiata (R+L; 205/231 voxel). In addition, smaller significantly different clusters were observed in the genu of the corpus callosum, the anterior limb of the internal capsule (R+L), and the superior corona radiata (R+L) (voxel involvement 19–84 voxels).

Significant changes in RD in patients between FU1 and FU2 occurred primarily in the same regions in which changes in FA were observed, including the genu (199 voxels) and the body of the corpus callosum (696 voxels), the anterior limb of the IC (R+L; 126/170 voxels), the anterior corona radiata (R+L; 174/483 voxels), and to a lesser extent the superior corona radiata (R+L), the external capsule (L), and the cingulum L (20–95 voxels).

### Correlation Between Relevant Diffusion Metrics With EDSS

In RRMS patients at baseline, significant negative correlations between DTI metrics and EDSS were mainly found in FA of the WM tracts that were significantly altered compared to HC (Table 6). Negative correlations with uncorrected *p* < 0.01 were detected in the left inferior cerebellar peduncle, the right cerebral peduncle, the left sagittal stratum, and the left external capsule. After applying a Bonferroni correction threshold of *p* < 0.002 to reduce the risk of false positives due to multiple testing, the negative correlations with FA in the left inferior cerebellar peduncle and the right cerebral peduncle remained significant. In patients with CIS, only a weak uncorrected inverse correlation was observed in the right posterior thalamic radiation (ρ = −0.286; *p* = 0.044).

**Table 6:** Spearman correlations between EDSS at baseline (BL) and at year 2 follow-up (FU2) and FA in regions with significant reductions (relative to healthy controls).

**Spearman correlation coefficients (*p*-value)**		**Baseline EDSS**	**Follow-up EDSS (year 2)**	
		**RRMS (*N* = 55)**	**Patients (*****N*** **=** **32)**	
	**MNI coordinates**	**FA BL vs. EDSS BL**	**FA BL vs. EDSS FU2**	**FA FU2 vs. EDSS FU2**
Genu of corpus callosum	(−3, −111, 5)		−0.370 (0.037)	
Middle cerebellar peduncle	(21, −43, −36)	−0.278 (0.040)		
Medial lemniscus R	(6, −37, −32)			−0.429 (0.014)
Medial lemniscus L	(−5, −37, −32)			−0.473 (0.006)
Inferior cerebellar peduncle L	(−6, −43, −52)	–**0.409 (0.002)**	−0.421 (0.016)	–**0.563 (0.001)**
Superior cerebellar peduncle R	(6, −33, −19)			−0.404 (0.022)
Cerebral peduncle R	(15, −116, −12)	–**0.409 (0.002)**	−0.457 (0.009)	–**0.637 (<0.001)**
Cerebral peduncle L	(−14, −116, −12)	−0.316 (0.019)		−0.462 (0.008)
Anterior limb of IC L	(−14, −114, 7)			−0.364 (0.040)
Posterior limb of IC L	(−23, −116, 13)	−0.316 (0.019)		
Retrolenticular part of IC L	(−27, −116, 6)	−0.319 (0.018)		
Anterior corona radiata R	(21, −110, 1)			−0.351 (0.049)
Superior corona radiata R	(27, −116, 24)	−0.322 (0.016)	−0.364 (0.040)	−0.392 (0.027)
Superior corona radiata L	(−26, −116, 24)		−0.368 (0.039)	−0.374 (0.035)
Sagittal stratum (include inf. long. fasc. and inf. front.-occip.fasc.) L	(−41, −29, −12)	−0.362 (0.007)		
External capsule R	(32, −113, −8)	−0.306 (0.023)		
External capsule L	(−31, −113, −8)	−0.361 (0.007)		
Cingulum (cingulate gyrus) L	(−7, −16, 36)		−0.365 (0.040)	
Fornix (cres)/R	(28, −116, −6)	−0.313 (0.020)	−0.393 (0.026)	−0.496 (0.004)
Fornix (cres)/L	(−27, −116, −6)	−0.295 (0.029)	−0.382 (0.031)	−0.415 (0.018)
Superior longitudinal fasciculus L	(64, −23, 30)			−0.434 (0.013)
Superior fronto-occipital fasciculus (ant. IC) R	(21, −114, 21)	−0.297 (0.028)		
**Cerebellum lobules**				
Left V	(−16, −51, −20)			−0.426 (0.015)
Right V	(13, −55, −17)		−0.458 (0.008)	−0.455 (0.009)
Left VI	(−14, −69, −22)		–**0.621 (<0.001)**	−0.427 (0.015)
Vermis VI	(1, −70, −21)			−0.510 (0.003)

*Baseline EDSS, correlations with FA in RRMS at BL; FU2 EDSS, correlations with FA at FU2 and with FA at BL for patients who experienced an EDSS change during the follow-up interval. Significant correlations (Bonferroni correction threshold p < 0.002) are marked in bold letters*.

*vs., versus; EDSS, expanded disability severity scale; BL, baseline; FU2, year 2 follow-up; FA, fractional anisotropy; L, left hemisphere; R, right hemisphere*.

Correlations with disability score (EDSS) were determined at follow-up in the subgroup of patients who received DTI at baseline, FU1, and FU2 and who experienced a change in EDSS during the follow-up period (*N* = 32) (Table 6).

Uncorrected negative correlations with *p* < 0.01 (Table 6) between EDSS at FU2 and altered FA values at baseline (patients compared to controls) were observed in the right cerebral peduncle and the cerebellar WM tracts of the right lobule V and left lobule VI. Of these, merely the negative correlation between FA in the left VI cerebellar lobule and EDSS remained significant after Bonferroni correction.

Regarding associations between EDDS at FU2 and FA at FU2, we found significant corrected negative correlations in the left inferior cerebellar peduncle and the right cerebral peduncle, while trends for further negative correlations (uncorrected *p* < 0.01) were observed in the left medial lemnisculus, left cerebral peduncle, right fornix cres., and the cerebellar lobules V (right) and vermis (VI) (Table 6).

Uncorrected correlations (*p* < 0.01) of RD alteration with EDSS were observed only at FU2 in the right cerebral peduncle, the left and right superior corona radiata, the left and right fornix cres., the left superior fasciculus, and cerebellar lobules I–IV right and VI left, which were not significant after Bonferroni correction.

## Discussion

In this study, brain WM alterations were investigated cross-sectionally and longitudinally in patients presenting with CIS or early MS at the onset of diagnosis to characterize the temporal dynamics of early microstructural WM changes and to assess indications of early axonal degeneration processes. Using a 3 Tesla MRI, differences in diffusion metrics between patients with CIS and MS in comparison with healthy controls were investigated using reproducible voxel-wise DTI-analysis (TBSS). The course of early effects on brain WM during the 2-year follow-up was characterized by longitudinal changes in FA and RD.

### Baseline Analysis at Disease Onset

Widespread reductions in FA involving most of the major brain WM tracts and the cerebellum were already observed at baseline, reflecting diffuse changes in WM integrity throughout the entire brains of individuals in the patient groups (Figure 2). Interestingly, the pattern of FA reductions in patients compared to controls was similar in the CIS and RRMS groups, except that the significant clusters were larger and the mean FA values were mostly lower in the RRMS group (Table 4), showing that this phenomenon occurs very early in the course of the disease, even before definitive diagnosis of MS. Our results agreed with recent observations of marked FA reductions in brain WM tracts in patients with CIS and more widespread disturbances in DTI metrics in patients with longstanding RRMS compared with patients with CIS (1, 3). Only a few regions, including the fornix (column and body) and the descending projection pathways (middle cerebellar and inferior cerebellar peduncle L and cerebral peduncle L), were exclusively altered in patients with RRMS. These results gave rise to the hypothesis that descending involvement of the infratentorial WM tracts may occur during the course of the disease. It is noteworthy that, at baseline, the widespread FA reductions observed in patients with CIS were not associated with EDSS, but FA alterations in patients with RRMS in the inferior and superior cerebellar peduncle were significantly correlated with EDSS, underlining the functional relevance of these descending projection pathways. The lack of correlation of EDSS with diffusion alterations in CIS agreed with previous findings (1).

Our study represents the first demonstration of associations between changes in FA at MS disease onset with future disability after 2 years, pointing to the predictive potential of these early WM alterations. In particular, FA reductions in the cerebral peduncle and cerebellar lobules V and VI were inversely correlated with EDSS at year two. These findings of changes in structural WM integrity at disease onset in MS and in CIS, and their associations with future disability, may support consideration of initiation of early therapy for both disease subtypes.

Correlations between EDSS and FA have been reported in previous studies on long-standing RRMS and in a recent meta-analysis, involving, in particular, the splenium of the corpus callosum and the pyramidal tracts (8, 9, 25). Recently, the impact of callosal diffusivity measures on disability progression in MS over 4 years was reported (18). In the present study on early MS, we detected weak correlations between FA reduction in the genu of the corpus callosum and future EDSS but no other significant associations involving callosal WM integrity changes (Table 6). These discrepancies may be a result of longer observation periods and differences in the MS subtypes included in these cited studies.

In agreement with the results of a recent study, we did not detect significant changes in RD in patients compared to healthy controls at baseline (1).

### Longitudinal TBSS Analysis (Follow-Up)

Longitudinal TBSS-based changes in diffusion metrics have rarely been investigated in early MS and CIS. In the present study, patterns of FA alterations in patients relative to healthy controls at years 1 and 2 were similar compared to the findings at baseline (Figure 2), with increasing cluster sizes beyond the first year. In accordance with previous results, the changes in DTI metrics between baseline and year 1 were not significant (26). This supported the hypothesis that microstructural WM damage is abundant at MS onset but evolves slowly, with delayed DTI changes during the first year of the disease. The progression of FA changes relative to HC in the descending pathways (cortico-spinal, cortico-cerebellar, pontine, and cerebellar tracts, see Table 4) at year 1 compared to year 2 suggested descending microstructural pathology in the course of the disease.

The clinical impact of fiber injury in these infratentorial descending structures was highlighted by significant inverse correlations between FA and EDSS, especially in the cerebral peduncles and cerebellar peduncle, and trends toward inverse correlation in cortico-pontine tracts and cerebellar WM tracts, at the second follow-up time point (Table 6).

RD alterations in patients compared to HC were undetectable at baseline and were only detected at FU1 in the corpus callosum and corona radiata, with progress of these differences at FU2. After 2 years, there was a considerable increase in the number of affected regions, with significant increases in RD in all major supratentorial WM tracts, the corpus callosum, and the cerebellum (Figure 2). Similar to FA, RD alterations became more evident in infratentorial structures at follow-up investigations, particularly in cerebellar structures, thus further supporting the hypothesis of descending microstructural WM degeneration during the course of MS.

At the second follow-up time point, there was a trend toward significant associations between increased RD and EDSS. RD alterations in the right cerebral peduncle, the left superior longitudinal fasciculus, and the left cerebellum lobules I–IV and VI showed correlations with EDSS (uncorrected *p* < 0.01). This supports the hypothesis put forward by Liu et al. that diffusion changes based on TBSS results and clinical correlations were mainly driven by increases in RD, and hence that RD predominantly reflected pathological changes in MS (8).

Longitudinal investigation between the first and second years of disease progression showed combined dynamic FA and RD changes in supratentorial structures, mainly in the corpus callosum and corona radiata. These dynamic longitudinal changes seemed to involve specific structures rather than diffuse widespread FA changes observed at baseline. Previous studies suggested that RD increases in MS may be indicative of axonal injury secondary to Wallerian degeneration (7). Still, other studies reported divergent results concerning the association of Wallerian degeneration with radial diffusivity or axial diffusivity (27, 28), and furthermore, axial and radial diffusivity measurements can be affected by the specific tissue type and geometry of the white matter tract (29). Thus, an association between Wallerian degeneration and longitudinal RD elevation in specific regions, as detected in our study, seems possible but should be confirmed by further studies. We hypothesize that pronounced longitudinal RD increases in specific structures may correspond to evolving axonal degeneration.

FA differences between patients and HC were present at disease onset. However, differences in FA and RD compared to HC in infratentorial and cerebellar structures by year 2 and combined dynamic FA and RD changes in supratentorial WM structures between the first and second years highlight the importance of changes in RD with regard to dynamic microstructural WM damage in the course of MS. Thus, our results suggested that FA alterations corresponded to diffuse underlying WM abnormalities in the brains of patients with MS in the earliest clinical phases, while RD changes in specific brain regions might represent later development of axonal degeneration during the clinical course of MS.

### Limitations

The main limitation of our study was the relatively small number of patients who received all follow-up examinations, which limited the statistical power of the longitudinal analyses. Nevertheless, in the TBSS analysis, we chose a conservative significance threshold, which ensured that only strong effects were reported and that the probability of type-1 errors was minimized. A second point was the lack of follow-up examinations for the control group, so longitudinal changes within the patient groups were assessed relative to baseline healthy control results. Since age-related physiological changes in DTI metrics may be present, the longitudinal differences in patients compared to baseline HC might be overestimated. Another limitation was the use of a DTI sequence with 32 gradient directions and a limited spatial resolution of 2.5 mm. Higher resolution and higher numbers of gradient directions and additional advanced imaging techniques such as NODDI (Neurite Orientation Dispersion and Density Imaging) or myelin water fraction imaging would allow for more detailed analysis of smaller fiber tracts and distinction between demyelination and axonal pathology in future MS studies (30, 31). Furthermore, lesions were not excluded from the start of the TBSS analysis (for example, by using masking tools), so in principle, the effect of lesions on the microstructural WM alterations could not be separated from diffuse, subacute changes. In the present study, the lesion load was low at baseline and the growth during the observation period was slow, so we estimated the effect of lesions by regarding the small overlapping areas of lesion distribution and FA or RD alterations.

We used the automatic LST-LGA algorithm on FLAIR weighted images for lesion segmentation without manually correcting for false-positive or false-negative findings. The lesion distributions might therefore have been overestimated in areas including the choroid plexus or the subcallosal ependymal rim. The lower sensitivity in the posterior fossa might have led to a loss of small cerebellar or brainstem lesions. The potential false positives probably have little impact on the results of the present study, because there was sparse overlap with the WM skeleton used in the TBSS analysis. False-negative brainstem or cerebellar lesions probably did not influence the results strongly, because the overall lesion load was low in this early MS and CIS cohort and mostly located supratentorially.

Further follow-up studies should include larger group sizes to enable assessment of differences between patients with CIS who convert to definitive MS and non-converters.

## Conclusions

Microstructural WM damage reflected by FA reduction was widespread throughout the entire brain immediately after disease onset in patients with MS and CIS. We demonstrated associations between FA at disease onset and future EDSS, suggesting the predictive value of FA reductions in the cerebellum and the cerebral peduncles with respect to disability.

Longitudinal changes in diffusion metrics and their clinical relevance in early MS were shown for the first time. The follow-up results during 2 years showed distinct temporal dynamics in DTI parameters, with constancy between baseline and the first follow-up (1 year) and increased changes in FA at the second follow-up time point (2 years). Furthermore, changes in RD, which were not significant at baseline, were detected at the first and moreover at the second follow-up time point.

Our results pointed to the value of increased RD as a marker of axonal injury, which also seemed to affect the descending projection pathways and cerebellar structures with corresponding EDSS relevance later in the course of disease progression. Dynamic combined FA and RD changes in longitudinally-investigated patients with MS were associated with specific structures, and increased RD may reflect progressing axonal degeneration.

In summary, TBSS investigations of diffusion metrics, at initial diagnosis and after two follow-up visits, showed subtle white matter pathology and the microstructural pathways involved in the progression of disease. Potentially predictive FA values at disease onset and increased RD values through the progression of MS are indicative of underlying WM pathology, mainly independent of local lesion load, which suggests that treatment of CIS and RRMS as early as possible may be warranted.

## Data Availability Statement

The datasets for this manuscript are not publicly available for reasons of patient confidentiality. Requests to access the datasets should be directed to the corresponding author RS (ruth.schneider@rub.de).

## Ethics Statement

The studies involving human participants were reviewed and approved by the local ethics committee of Ruhr-University Bochum (Approval No. 3714-10). The patients/participants provided their written informed consent to participate in this study.

## Author Contributions

RS: design of the work, acquisition, analysis and interpretation of data for the work, drafting and critical revision of the work for important intellectual content, final approval of the version to be published, and agreement to be accountable for all aspects of the work. EG: analysis and interpretation of data for the work and final approval of the version to be published. CA: acquisition, analysis and interpretation of data for the work, and final approval of the version to be published. RG: conception of the work, interpretation of data for the work, critical revision of the work for important intellectual content, and final approval of the version to be published. CL: conception and design of the work, interpretation of data for the work, critical revision of the work for important intellectual content, and final approval of the version to be published. BB: acquisition, analysis and interpretation of data for the work, drafting and critical revision of the work for important intellectual content, final approval of the version to be published, and agreement to be accountable for all aspects of the work.

### Conflict of Interest

RS has received consulting and speakers honoraria from Biogen Idec GmBH and Roche Pharma AG and has received research scientific grant support from Novartis Pharma. RG has received compensation for serving as a consultant or speaker from Bayer HealthCare, Biogen Idec, Merck Serono, Novartis, and Teva Neuroscience; he, or the institution he works for, has received research support from Bayer HealthCare, Biogen Idec, Merck Serono, Novartis, and Teva Neuroscience; he has also received honoraria as a Journal Editor from SAGE and Thieme Verlag. CL has received consulting and speaker's honoraria from Biogen Idec, Bayer Schering, Daiichi Sanykyo, Merck Serono, Novartis, Sanofi, Genzyme, and TEVA. The remaining authors declare that the research was conducted in the absence of any commercial or financial relationships that could be construed as a potential conflict of interest.
