# Supplementary material for: Temporal Dynamics of Diffusion Metrics in Early Multiple Sclerosis and Clinically Isolated Syndrome: A 2-Year Follow-Up Tract-Based Spatial Statistics Study
Source: Front Neurol. 2019 Nov 5;10:1165. doi: 10.3389/fneur.2019.01165 (PMC6848258; doi:10.3389/fneur.2019.01165)

Supplementary material

Table S1 New clusters of significant FA reduction (patients relative to HC) at year 2 predominantly in infratentorial tracts: those anatomical regions which were not affected at year 1, but which were markedly affected at year 2 are shown (ordered according to cluster size). Mean and standard deviations of FA in significant clusters at year 2 show lower FA in patients than in HC.

**Region** (mni coordinates: x,y,z)		voxels year 1	voxels year 2	**FA (mean ± SD)** Patients at year 2	**FA (mean ± SD)** HC
N		83	43	43	49
Middle cerebellar peduncle	(21,-43,-36)	-	992	0.83 ± 0.03	0.86 ± 0.02
Corticospinal tract R	(8,-116,-29)	-	203	0.84 ± 0.05	0.86 ± 0.03
Superior longitudinal fasciculus L	(-36,-23,30)	-	176	0.71 ± 0.05	0.74 ± 0.05
Medial lemniscus R	(6,-37,-32)	-	87	0.88 ± 0.04	0.89 ± 0.02
Superior cerebellar peduncle R	(6,-33,-19)	-	82	0.86 ± 0.04	0.87 ± 0.03
Inferior cerebellar peduncle L	(-6,-43,-52)	-	78	0.82 ± 0.03	0.84 ± 0.03
Inferior cerebellar peduncle R	(7,-43,-52)	-	77	0.72 ± 0.04	0.75 ± 0.03
Pontine crossing tract	(1,-29,-32)	-	76	0.78 ± 0.06	0.81 ± 0.04
Superior cerebellar peduncle L	(-5,-33,-19)	-	75	0.86 ± 0.04	0.88 ± 0.04
Medial lemniscus L	(-5,-37,-32)	-	54	0.82 ± 0.04	0.84 ± 0.03
Uncinate fasciculus L	(-33,-114,-14)	-	43	0.74 ± 0.08	0.77 ± 0.07
Corticospinal tract L	(-7,-116,-29)	-	15	0.73 ± 0.11	0.76 ± 0.07
Superior fronto-occipital fasciculus R	(21,-114,21)	-	5	0.89 ± 0.06	0.91 ± 0.05
**Cerebellum lobules** (mni coordinates: x,y,z)		-			
Right I-IV	(7,-46,-17)	-	428	0.36 ± 0.03	0.39 ± 0.03
Left I-IV	(-6,-46,-18)	-	383	0.34 ± 0.03	0.37 ± 0.03
Right V	(13,-55,-17)	-	383	0.37 ± 0.03	0.40 ± 0.02
Left V	(-16,-51,-20)	-	357	0.37 ± 0.03	0.40 ± 0.02
Vermis VI	(1,-70,-21)	-	235	0.42 ± 0.04	0.44 ± 0.03
Left VI	(-14,-69,-22)	-	158	0.31 ± 0.03	0.33 ± 0.03

Abbreviations: FA fractional anisotropy, SD standard deviation, HC healthy controls, N number of participants, L left hemisphere, R right hemisphere.

Table S2 Significant RD changes in patients compared to healthy controls (HC) in the follow-up investigations in those anatomical regions which were not or barely affected at year 1, but which were markedly affected at year 2. The sizes of significant clusters for each region are reported as voxel numbers in cluster (in descending order). An increase of voxel number represents larger cluster sizes at year 2 compared to year 1. Mean and standard deviations of RD in significant clusters at year 2 show higher RD in patients than in HC.

**Region** (coordinates: x,y,z)		voxel no. in cluster		**increase of voxel no.**	**RD (10^{-3} mm^2/s) (mean ± SD)**	
		year 1	year 2		Patients at year 2	HC
N		83	43		43	49
Body of corpus callosum	(-5,-116,25)	1074	2786	1712	0.082 ± 0.068	0.039 ± 0.033
Anterior corona radiata R	(21,-110,1)	-	1326	1326	0.184 ± 0.034	0.161 ± 0.022
Genu of corpus callosum	(-3,-111,5)	-	1127	1127	0.021 ± 0.054	0.007 ± 0.033
Anterior corona radiata L	(-20,-110,1)	-	1121	1121	0.181 ± 0.037	0.161 ± 0.021
External capsule L	(-31,5,-8)	-	1080	1080	0.212 ± 0.051	0.182 ± 0.034
Posterior thalamic incl.optic radiation L	(-32,-62,1)	5	999	994	0.152 ± 0.038	0.117 ± 0.026
Posterior thalamic incl.optic radiation R	(33,-62,1)	-	969	969	0.143 ± 0.038	0.111 ± 0.030
Splenium of corpus callosum	(5,-37,16)	163	1074	911	0.055 ± 0.057	0.018 ± 0.024
External capsule R	(32,-113,-8)	-	850	850	0.232 ± 0.047	0.204 ± 0.036
Superior corona radiata L	(-26,-116,24)	96	902	806	0.164 ± 0.035	0.140 ± 0.024
Posterior corona radiata R	(26,-27,27)	4	769	765	0.207 ± 0.039	0.177 ± 0.028
Superior corona radiata R	(27,-116,24)	6	713	707	0.163 ± 0.029	0.142 ± 0.023
Posterior corona radiata L	(-25,-27,27)	84	651	567	0.215 ± 0.034	0.188 ± 0.026
Superior longitudinal fasciculus R	(37,-23,30)	-	502	502	0.179 ± 0.035	0.164 ± 0.017
Retrolenticular part of IC R	(29,-116,6)	-	456	456	0.151 ± 0.033	0.130 ± 0.025
Sagittal stratum R	(42,-29,-12)	-	399	399	0.167 ± 0.041	0.145 ± 0.026
Superior longitudinal fasciculus L	(-36,-23,30)	-	337	337	0.181 ± 0.030	0.165 ± 0.021
Retrolenticular part of IC L	(-27,-116,6)	-	318	318	0.151 ± 0.032	0.131 ± 0.029
Sagittal stratum L	(-41,-29,-12)	-	294	294	0.192 ± 0.042	0.162 ± 0.024
Anterior limb of IC L	(-14,-114,7)	-	280	280	0.077 ± 0.058	0.053 ± 0.030
Cingulum (cingulate gyrus) R	(7,6,33)	-	216	216	0.162 ± 0.045	0.139 ± 0.025
Cingulum (cingulate gyrus) L	(-7,-16,36)	-	201	201	0.158 ± 0.054	0.133 ± 0.027
Fornix (cres) / R	(28,-116,-6)	-	195	195	0.176 ± 0.055	0.142 ± 0.034
Fornix (column and body	(1,-115,16)	-	189	189	0.586 ± 0.220	0.449 ± 0.160
Anterior limb of IC R	(-14,-116,-12)		183	183	0.087 ± 0.036	0.062 ± 0.033
Fornix (cres) / L[117,100,66]	(-27,-116,-6)	-	174	174	0.151 ± 0.045	0.126 ± 0.031

Cingulum (hippocampus) R(66,101,53)	(24,-116,-19)	-	136	136	0.158 ± 0.041	0.138 ± 0.045
**Cerebellum lobules**(coordinates: x,y,z)						
Left V	(-16,-51,-20)	-	177	177	0.482 ± 0.087	0.438 ± 0.049
Left I-IV	(-6,-46,-18)	-	74	74	0.660 ± 0.150	0.585 ± 0.110
Right I-IV	(7,-46,-17)	-	67	67	0.642 ± 0.120	0.580 ± 0.120
Left VI(	(-14,-69,-22)	-	63	63	0.465 ± 0.089	0.432 ± 0.049
Right V	(13,-55,-17)	-	38	38	0.590 ± 0.100	0.537 ± 0.086
Left Crus I	(-42,-65,-33)	-	14	14	0.538 ± 0.160	0.495 ± 0.140

Abbreviations: RD radial diffusivity SD standard deviation, HC healthy controls, N number of participants, L left hemisphere, R right hemisphere

Supplemental Figure S1 Lesion distribution (hyperintense FLAIR lesions) in patients who received MRI at baseline and year 2 (N=43) overlayed on a T1-template (MNI-coordinate space) and the FA-skeleton (yellow): blue overlay represents the mean lesion distribution at baseline, red/orange overlay: mean lesion distribution at year 2. The mean lesion maps were thresholded at a level of 0.1; Z: mni-coordinates of slice positions.

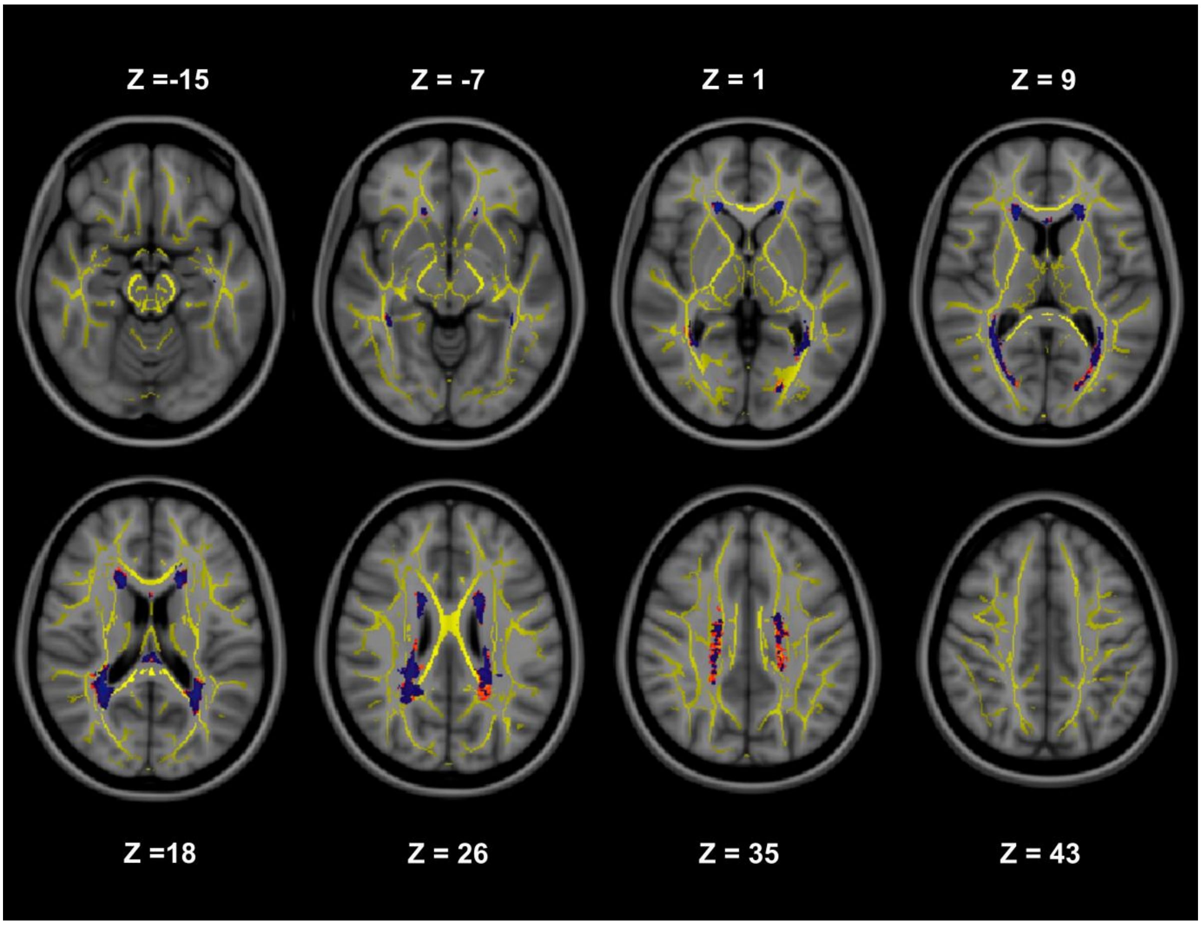

Supplement: Supplementary file 1 [file Data_Sheet_1.PDF]
